# Germ plasm localisation of the HELICc of Vasa in *Drosophila*: analysis of domain sufficiency and amino acids critical for localisation

**DOI:** 10.1038/srep14703

**Published:** 2015-09-30

**Authors:** Szu-Chieh Wang, Hao-Jen Hsu, Gee-way Lin, Ting-Fang Wang, Chun-che Chang, Ming-Der Lin

**Affiliations:** 1 Department of Molecular Biology and Human Genetics, Tzu-Chi University, Hualien, Taiwan; 2 Department of Life Science, Tzu-Chi University, Hualien, Taiwan; 3 Laboratory for Genetics and Development, Department of Entomology/Institute of Biotechnology, College of Bioresources and Agriculture, National Taiwan University, Taipei, Taiwan; 4 Research Center for Developmental Biology and Regenerative Medicine, National Taiwan University, Taipei, Taiwan; 5 Genome and Systems Biology Degree Program, National Taiwan University and Academia Sinica, Taipei, Taiwan

## Abstract

Formation of the germ plasm drives germline specification in *Drosophila* and some other insects such as aphids. Identification of the DEAD-box protein Vasa (Vas) as a conserved germline marker in flies and aphids suggests that they share common components for assembling the germ plasm. However, to which extent the assembly order is conserved and the correlation between functions and sequences of Vas remain unclear. Ectopic expression of the pea aphid Vas (ApVas1) in *Drosophila* did not drive its localisation to the germ plasm, but ApVas1 with a replaced C-terminal domain (HELICc) of *Drosophila* Vas (DmVas) became germ-plasm restricted. We found that HELICc itself, through the interaction with Oskar (Osk), was sufficient for germ-plasm localisation. Similarly, HELICc of the grasshopper Vas could be recruited to the germ plasm in *Drosophila*. Nonetheless, germ-plasm localisation was not seen in the *Drosophila* oocytes expressing HELICcs of Vas orthologues from aphids, crickets, and mice. We further identified that glutamine (Gln) 527 within HELICc of DmVas was critical for localisation, and its corresponding residue could also be detected in grasshopper Vas yet missing in the other three species. This suggests that Gln527 is a direct target of Osk or critical to the maintenance of HELICc conformation.

In Metazoans, germ cell specification is critical for establishing the germline lineage. For acquiring germline identity, cells can recruit germline determinants from maternal germ plasm, typically preformed in an uncellularised egg chamber, or receive signals from neighbouring cells to induce the expression of germline genes^1^. No matter how germ cells are specified, the preferential expression of *vasa* (*vas*) mRNA or Vas protein has become a hallmark of primordial germ cells as well as gonadal germ cells. *vas* encodes an ATP-dependent RNA helicase of the DEAD (Asp-Glu-Ala-Asp)-box family. It was initially identified in the fruit fly *Drosophila melanogaster* in late 1980s^2^,^3^,^4^ and has since been found in many other established and emerging animal models^5^. Various conserved functions for Vas protein have been proposed, including translationally regulating other germline genes, interacting with other germline components, and involvement with aspects of RNA metabolism such as piRNA biogenesis^6^.

During *Drosophila* oogenesis, maternal Vas is localised to the germ (pole) plasm in the posterior end of developing oocytes. The localisation of Vas is found to be dependent on the predeposition of Oskar (Osk) protein in the posterior end of the oocyte after stage 9^7^,^8^. After fertilisation, Vas, regardless of whether it is maternal or zygotic, is preferentially expressed in the germ cells throughout embryogenesis^7^,^8^. Embryos produced by *vas* hypomorphic mutants fail to form pole cells and abdominal segments^3^, and homozygous *vas*-null females exhibit aberrant ovarioles with atrophied germaria, fewer egg chambers, or mispatterned oocytes^9^. As an RNA helicase, Vas can promote the translation of *osk* and *nanos* in the germ plasm^10^,^11^,^12^,^13^. Moreover, the Vas activity is found to be regulated by meiotic checkpoint pathway to control the *gurken* translation in the oocyte^14^. Although how Vas regulates the translation of specific mRNAs remains unclear, the direct interaction between Vas and the general translation initiation factor eIF5B (also known as dIF2) is reckoned as a potential cause^15^,^16^. As such, Vas–eIF5B interaction can positively regulate the translation of *gurken* and *mei-P26* for anteroposterior/dorsoventral patterning in oocytes^15^ and germline stem cell differentiation^16^, respectively.

*Drosophila* Vas, abbreviated as DmVas to distinguish it from other Vas proteins mentioned in this study, is composed of 661 amino acids. Similar to other DEAD-box helicases, DmVas contains conserved helicase signature motifs that compose the ‘helicase core’, but sequences flanking this region are less conserved (Supplementary Fig. S1A). Regarding the connections between sequences and functions, we summarize the previous findings as follows: (1) RGG (Arg-Gly-Gly) repeats, ranging from N-terminal residues 17–165, play a potential role in assisting RNA binding^17^,^18^; (2) the DINNN motif (residues 184–188) is a binding site of the SOCS-box protein Gustavus, a protein that can stabilise DmVas accumulation in the germ plasm^19^,^20^; (3) the ‘helicase core’ comprises an DEAD-like helicases N-terminal domain (DEXDc; residues 233–454) and a helicase superfamily C-terminal domain (HELICc; residues 463–621) for hydrolysing ATP and driving RNA duplex unwinding^21^,^22^; and (4) three C-terminal amino acids (residues 616–618) of the helicase core are critical for the interaction of DmVas with eIF5B^15^. Furthermore, studies on EMS-induced mutations have shown that point mutations such as V465M, S518F, H520Y, and G587E within the helicase core abolish the germ plasm localisation of DmVas whilst still preserving the helicase activity^23^. This suggests that the helicase core is not only critical for RNA unwinding, the helicase activity itself, but also required for the localisation of DmVas to the germ plasm.

At first, we expressed ApVas1, a pea aphid orthologue of DmVas, in *Drosophila* to examine whether it could be posteriorly localised to the germ plasm. Given that ApVas1 is a germ plasm component in the pea aphid^24^,^25^, this experiment can shed light on whether machineries for anchoring DmVas/ApVas1 to the germ plasm are conserved. Unlike DmVas, ApVas1 was not posteriorly localised in the *Drosophila* oocyte, suggesting that the lack of specific sequences for being restricted to the germ plasm is the cause. Accordingly, we employed domain swapping between DmVas and ApVas1, finding that the HELICc domain of DmVas was sufficient for being localised to the germ plasm in an Osk-dependent manner. Furthermore, the first ten amino acids and Gln527 in HELICc were identified as key residues required for the germ plasm localisation of DmVas. Molecular dynamics (MD) simulations further demonstrated that these residues played a decisive role in maintaining the integrity of the HELICc structure. Sequences located N-terminal to the helicase core, by contrast, were found essential to pole cell formation and posterior development. Altogether, our results unveil critical connections between sequences and functions of DmVas during oogenesis and early embryogenesis.

## Results

### Ectopic expression of ApVas1 in *Drosophila* oocytes

**Figure 1:**
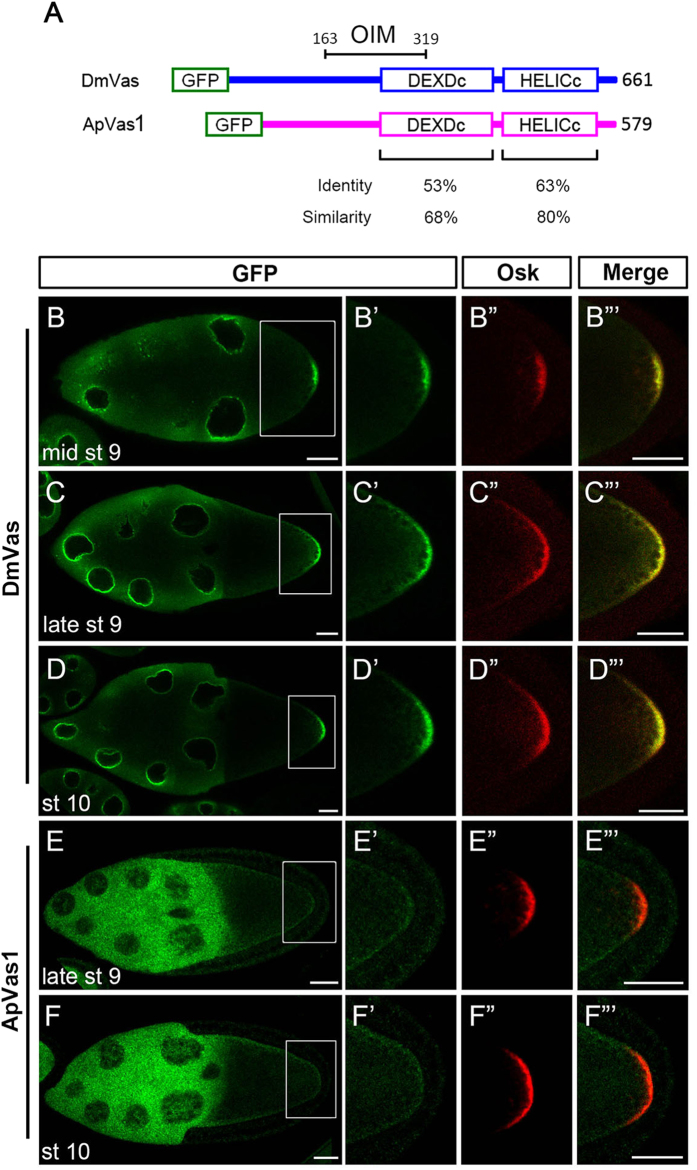
ApVas1 was not colocalised with Oskar (Osk) to the posterior germ plasm of *Drosophila* oocyte. (**A**) Schematic alignment of *Drosophila* Vasa (DmVas) and the pea aphid Vasa (ApVas1). Open boxes show the green fluorescent protein (GFP) tag, the DEAD-like helicases superfamily (DEXDc), and helicase superfamily C-terminal (HELICc) domains. Sequence identity and similarity are highlighted beneath the domain boxes. Location of the Osk interacting motif (OIM) of DmVas: amino acids 163–319. (**B–D**) Posterior localisation of GFP-DmVas in the oocyte of Stage-9–10 egg chambers. (**E**,**F**) Expression of GFP-ApVas1 in the late Stage-9 and Stage-10 egg chambers. Posterior localisation of GFP-ApVas1 was not identified. (**B’–F’**) Magnification of the insets shown in (**B**–**F**). (**B”–F”**) Posterior localisation of Osk. (**B”’–F”’**) Merged images. The egg chambers were double stained using antibodies against GFP (green) and Osk (red). In all panels, anterior is to the left and posterior is to the right. Scale bars, 25 *μ*m.

Alignment of DmVas and ApVas1, which are germline markers in *Drosophila* and the pea aphid, respectively, displays highly conserved features in their helicase core domains DEXDc and HELICc^21^ (Fig. 1A; Supplementary Fig. S1A). To understand the extent of similarity in the functions of ApVas1 and DmVas, we specifically expressed green fluorescent protein (GFP)-ApVas1 in the *Drosophila* female germline by using a maternal *tubulin* 67*c* promoter^26^. GFP-DmVas expression was driven by the same promoter and served as a positive control. As expected, the posterior localisation of DmVas to the germ plasm was observed from mid-stage 9 of oogenesis^8^,^27^, right after Osk could first be detected in the posterior pole of the oocytes^28^,^29^,^30^ (Fig. 1B–B”’). Colocalisation of DmVas and Osk to the germ plasm became even more prominent in the egg chambers at late stage 9 (Fig. 1C–C”’) and stage 10 (Fig. 1D–D”’). However, during the same period of oogenesis, ApVas1 was not colocalised with Osk in the posterior germ plasm (Fig. 1E–E”’”’). Instead, we could only visualise a weak expression of ApVas1 in the lateral cortex of the oocytes (Fig. 1E,F).

### Analysis of sequences required for the posterior localisation of DmVas to the germ plasm

**Figure 2:**
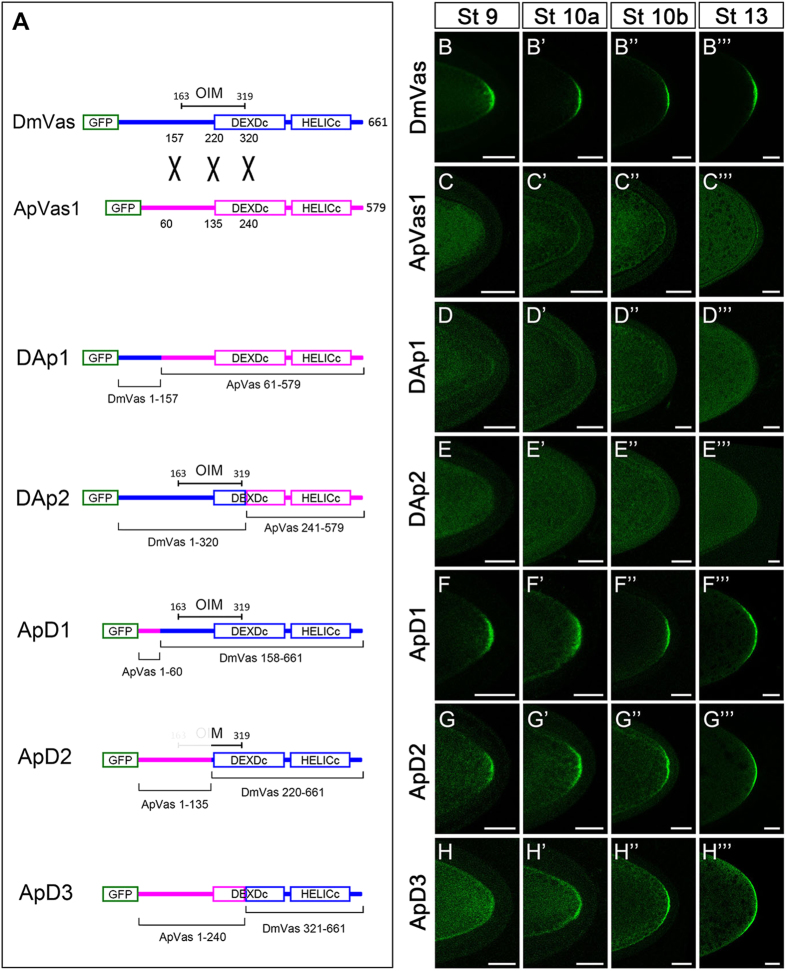
Localisation of chimeric proteins composed of *Drosophila* Vasa (DmVas) and pea aphid Vasa (ApVas1) in the oocyte. (**A**) Schematic illustration of domain swapping between DmVas and ApVas1. Blue and pink colours represent sequences derived from DmVas and ApVas1, respectively. The same colour codes are used in the other Fig. of this paper. (**B–H’”**) Localisation analysis of the green fluorescent protein (GFP)-tagged chimeric proteins in the oocytes during oogenesis from stages 9–13. (**B–B”’**) Posterior localisation of GFP-DmVas: a positive control. (**C–C”’**) GFP-ApVas1: posterior localisation was not detected. (**D–D”’**) GFP-DAp1 (DmVas^1–157^ + ApVas1^61–579^; Osk interacting motif (OIM) was not included): posterior localisation was not detected. (**E–E”’**) GFP-DAp2 (DmVas^1–320^ + ApVas1^241–579^; OIM was included): posterior localisation was not detected. (**F–F”’**) GFP-ApD1 (ApVas1^1–60^ + DmVas^158–661^; OIM was included): posterior localisation was detected. (**G–G”’**) GFP-ApD2 (ApVas1^1–135^ + DmVas^220–661^; OIM was partially truncated): posterior localisation was detected. (**H–H”’**) GFP-ApD3 (ApVas1^1–240^ + DmVas^321–661^; OIM was not included): posterior localisation became prominent from Stage 10a onwards. In all panels, anterior is to the left and posterior is to the right. Scale bars, 25 *μ*m.

In *Drosophila*, the germ plasm localisation of Vas is dependent on the pre-deposition of Osk in the posterior pole of the oocyte^7^,^8^. By using yeast two-hybrid and GST pull-down assays, the DmVas sequences required for the Osk–Vas interaction had been found to span amino acids 163–319^31^,^32^; here we refer to this region as the Osk interaction motif (OIM). Such physical interaction between Osk and DmVas has been considered essential for the germ plasm localisation of DmVas. In order to determine whether the lack of OIM sequence is responsible for the failure of posterior localisation of ApVas1 in *Drosophila* oocyte, we performed domain-swapping analyses replacing various lengths of N-terminal ApVas1 sequences with N-terminal DmVas sequences (Fig. 2A). DAp1 is a chimeric protein formed by replacing the first 60 amino acids of ApVas1 with the N-terminal 157 amino acids of DmVas (Fig. 2A). As expected, unlike DmVas (Fig. 2B–B”’), DAp1 was not localised to the posterior germ plasm (Fig. 2D–D”’) the same as ApVas1 (Fig. 2C–C”’). However, to our surprise, DAp2 whose sequence contained an intact OIM in the N-terminal 320 residues of DmVas (DmVas^1–320^) was still not posteriorly localised to the oocyte (Fig. 2E–E”’).

In addition to DAp1 and DAp2, we synthesized ApD1, ApD2, and ApD3, which were chimeric proteins with ApVas1 at the N-termini and DmVas at the C-termini (Fig. 2A). We found that both ApD1 (with an intact OIM) and ApD2 (with a partial OIM sequence) could be directed to the germ plasm from stages 9 to 13 of oogenesis (Fig. 2F–F”’”’). Moreover, ApD3, though without any OIM sequence, could be localised to the germ plasm (Fig. 2H’–H”’). Nevertheless, we noticed that the posterior localisation of ApD2 (Fig. 2G) was not as prominent as that of ApD1 (Fig. 2F) at stage 9 of oogenesis. Further, ApD3 began to be enriched in the posterior pole of the oocyte only after stage 10 (Fig. 2H’–H”’). These results suggest that OIM sequence is not sufficient to direct the posterior localisation of DmVas but instead is required for the efficient accumulation of DmVas to the germ plasm at stage 9.

### The HELICc of DmVas is sufficient to be localised to the germ plasm and nuage

**Figure 3:**
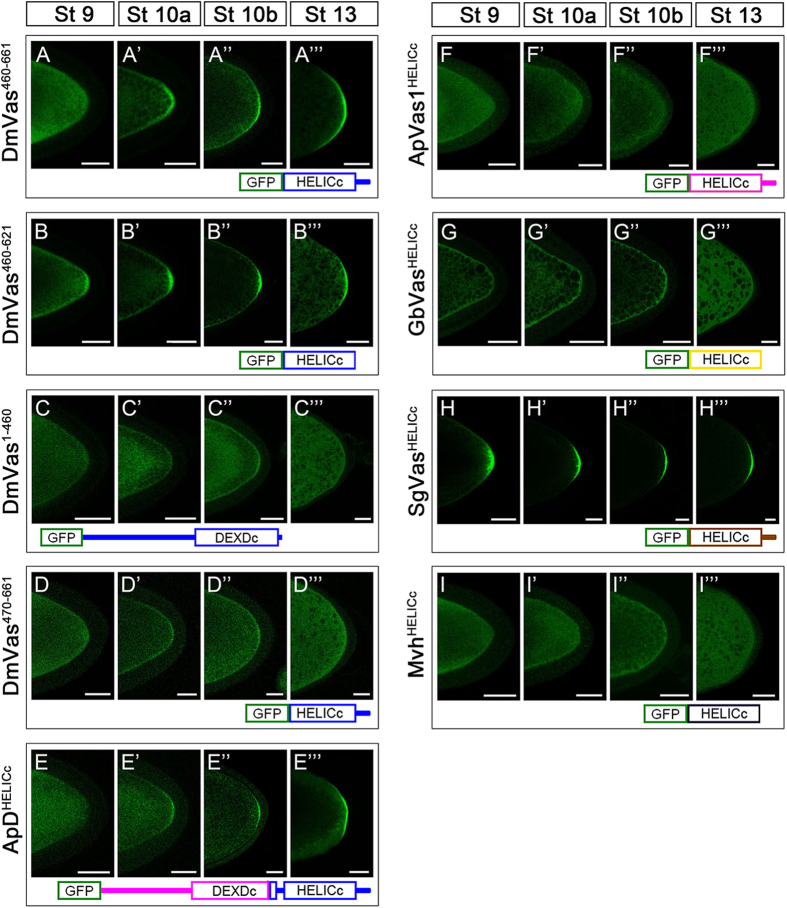
Localisation analysis of truncated *Drosophila* Vasa (DmVas) proteins, chimeric Vas proteins, and helicase superfamily C-terminal domains (HELICc) from other animal species. All Vas proteins were green fluorescent protein (GFP)-tagged and analyses were performed in egg chambers from Stages 9–13 of oogenesis. Each panel shows the posterior half of the oocyte. (**A–A”’**) GFP-DmVas^460–661^: sequence N-terminal to HELICc was truncated; (**B–B”’**) GFP-DmVas^460–621/HELICc^ (abbreviated as DmVas^460–621^): a sole HELICc. Posterior localisation of DmVas^460–661^ and DmVas^460–621/HELICc^ was detected; (**C–C”’**) GFP-DmVas^1–460^: a C-terminal truncated DmVas polypeptide without the HELICc and C-terminal sequences. (**D–D”’**) GFP-DmVas^470–661^: an N-terminal truncated DmVas polypeptide without the N-terminal sequence, DEXDc, and 10 residues in the N-terminus of HELICc. Posterior localisation of DmVas^1–460^ and DmVas^470–661^ was not detected; (**E–E”’**) GFP-ApD^HELICc^: a ApVas1–DmVas chimeric protein in which the N-terminal sequence and most of the DEXDc domain sequences of DmVas were replaced by those from ApVas1. Posterior localisation was detected; (**F–F”’** to **I–I”’**) GFP-tagged HELICc of Vas orthologs from the pea aphid (F–F”’, GFP-ApVas1^HELICc^), cricket (G–G”’, GFP-GbVas^HELICc^), grasshopper (H–H”’, GFP-SgVas^HELICc^), and mouse (I–I”’, GFP-Mvh^HELICc^). Posterior localisation could be detected only in the egg chamber expressing SgVas^HELICc^ (H–H”’). In all panels, anterior is to the left and posterior is to the right. Scale bars, 25 *μ*m.

**Figure 4:**
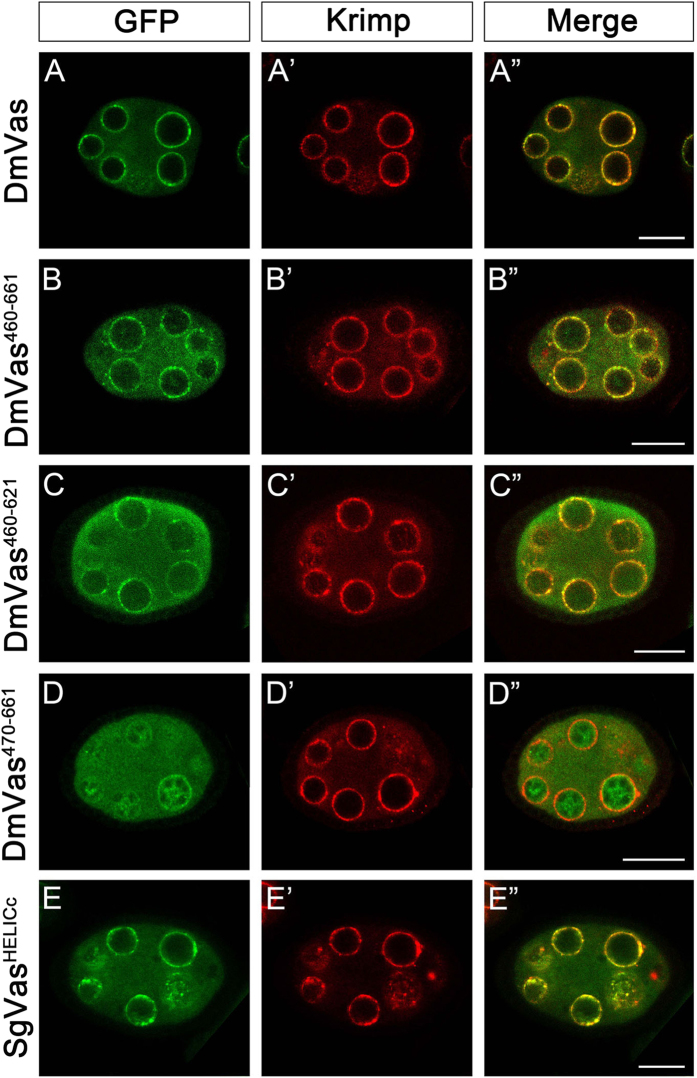
Localisation analysis of truncated *Drosophila* Vasa (DmVas) proteins and the helicase superfamily C-terminal domain (HELICc) of grasshopper Vasa (SgVas) in the nuage. Stage-5–6 egg chambers expressing green fluorescent protein (GFP)-tagged Vas proteins were double stained using the anti-GFP (green) and anti-Krimp antibodies (red). (**A–E**) GFP staining (green); (**A’–E’**) Krimp staining (red); (**A”–E”**) Merged images. For protein features, see Fig. 3. (**A**–**A”’**) Colocalisation of GFP-DmVas and Krimp to nuages surrounding the nuclear envelope of nurse cells: a positive control. (**B–B”’**,**C–C”’**) GFP-DmVas^460–661^, and GFP-DmVas^460–621^: both of these two DmVas truncations contain an intact HELICc sequence. Colocalisation was identified. (**D–D”’**) GFP-DmVas^470–661^, a truncated HELICc sequence of DmVas with a 10-amino acid deletion in its N-terminus, was not colocalised with Krimp. (**E–E”’**) GFP-SgVas^HELICc^: HELICc of grasshopper SgVas. Colocalisation with Krimp was identified. In all panels, anterior is to the left and posterior is to the right. Scale bars, 20 *μ*m.

Given that ApD3 without an OIM could still be localised to the germ plasm (Fig. 2H’–H”’), we inferred that sequences within DEXDc or HELICc may contribute to the posterior localisation of DmVas. To test this inference, we further truncated all the amino acids N-terminal to HELICc and found that DmVas could still be localised to the germ plasm from stage 10 onwards (DmVas^460–661^, Fig. 3A–A”’). Similar patterns could also be observed in DmVas containing a single HELICc (DmVas^460–621/HELICc^, Fig. 3B–B”’). By contrast, a DmVas protein whose HELICc and sequences C-terminal to HELICc were all truncated was not posteriorly localised (DmVas^1–460^, Fig. 3C–C”’). We further found that deletion of the residues 460–469 in HELICc disabled the posterior localisation (DmVas^470–661^, Fig. 3D–D”’). When sequences located N-terminal to HELICc were replaced with corresponding ApVas1 sequences, posterior localisation still occurred (ApD^HELICc^, Fig. 3E–E”’). Altogether, these results suggest that residues 460–469 within HELICc are critical for the posterior localisation of DmVas to the germ plasm. However, we did not observe germ plasm localisation of HELICc derived from ApVas1 (ApVas1^HELICc^, Fig. 3F–F”’), cricket *Gryllus bimaculatus* Vasa (GbVas^HELICc^, Fig. 3G–G”’), or the mouse Vas homolog protein (Mvh^HELICc^, Fig. 3I–I”’) in the *Drosophila* oocytes. These HELICc proteins, instead, were uniformly distributed in the cytoplasm. Although a low level of localisation of the GbVas^HELICc^ was identified in the cortex of the oocyte, it was not particularly enriched to the germ plasm (Fig. 3G’”). The HELICc of grasshopper *Schistocerca gregaria* Vasa (SgVas^HELICc^), surprisingly, was localised to the germ plasm of *Drosophila* oocyte (Fig. 3H–H”’), yet in *S*. *gregaria* a maternal germ plasm expressing SgVas had not been identified^33^. In addition to the germ plasm, DmVas is known to be localised to the nuage, an electron-dense structure restricted to the nuclear periphery of nurse cells^8^,^23^,^34^,^35^. Here, we used Krimp as a nuage marker to investigate whether truncated DmVas proteins could be localised to the nuage^36^. We found that DmVas^460–661^ (Fig. 4B–B”) and DmVas^460–621/HELICc^ (Fig. 4C–C”), both of which containing intact HELICc, could be colocalised with Krimp to the nuage as full length DmVas (Fig. 4A–A”). By contrast, the shortened HELICc lacking the residues 460–469 was mis-localised to the nucleus and did not exhibit nuage localisation in the perinuclear region (DmVas^470–661^, Fig. 4D–D”). Similar to DmVas, HELICc of SgVas was restricted to the nuage and colocalised with Krimp (SgVas^HELICc^, Fig. 4E–E”). These results suggest that the HELICc domain could also encompass amino acids critical for nuage localisation.

### Osk–HELICc interaction facilitates the germ plasm localisation of DmVas

**Figure 5:**
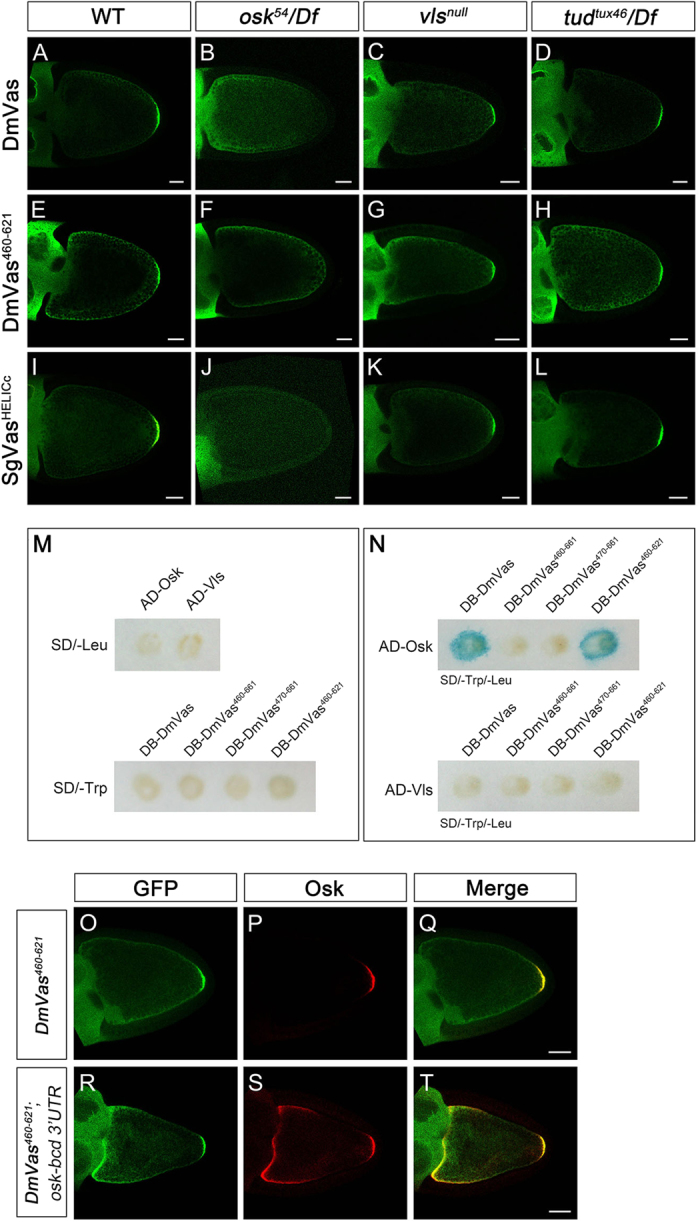
Oskar (Osk) interacts with the helicase superfamily C-terminal domain (HELICc) of *Drosophila* Vasa (DmVas) *in vivo* and *in vitro*. (**A–L**) Localisation analyses of (**A**–**D**) green fluorescent protein (GFP)-DmVas, (**E**–**H**) GFP-Vas^460–621/HELICc^, and (**I**–**L**) GFP-SgVas^HELICc^ were performed in egg chambers at Stage 10 by immunostaining with the anti-GFP antibody. Genetic backgrounds: (**A**,**E**,**I**) Wild-type; (**B**,**F**,**J**) *osk* mutant with the genotype *osk*^*54*^/*Df(3R)pXT103*; (**C**,**G**,**K**) *vls* null mutant with the genotype *Df(2L)Pr2b,P[barren+]*/*Df(2L)be408*; (**D**,**H**,**L**) *tud* mutant with the genotype *tud*^*tux46*^/*Df(2R)PF1*. All the 3 GFP-tagged Vas proteins could be localised to the posterior germ plasm, except in the *osk* mutant background. (**M**,**N**) Yeast two-hybrid analysis performed using the β-galactosidase colony lift filter assay. (**M**) None of the singly transformed ‘bait’ and ‘prey’ plasmids could induce the expression of the *lacZ* reporter. (**N**) Osk could interact with full-length DmVas and DmVas^460–621/HELICc^. (**O–T**) Stage-10 egg chambers were stained with the anti-GFP (green) and anti-Osk antibodies (red). (**Q**,**T**) Merged images. (**O**–**Q**) GFP-DmVas^460–621/HELICc^ was colocalised with Osk in the germ plasm. (**R**–**T**) In the egg chambers coexpressing the GFP-DmVas^460–621/HELICc^ and *osk-bcd 3′UTR* transcripts, the GFP-DmVas^460–621/HELICc^ was colocalised with Osk in the anterior and posterior poles of the oocyte. In the panels with egg chambers (**A**–**L**,**O**–**T**), anterior is to the left and posterior is to the right. The DmVas^460–621/HELICc^ is abbreviated as DmVas^460–621^ in all the panels. Scale bars, 25 *μ*m.

To address how HELICc was localised to germ plasm, we investigated whether its posterior localisation in the oocyte was dependent on Osk. In wild-type egg chambers, both the full-length DmVas (Fig. 5A) and the sole HELICc (DmVas^460–621/HELICc^, Fig. 5E) were localised to the germ plasm. Similar to full-length DmVas as previously reported^37^ (Fig. 5B), the posterior localisation of HELICc was not observed in the *osk* mutant egg chambers (Fig. 5F). As both Valois (Vls)^38^ and Tudor (Tud)^39^ are two known downstream components of Osk, we further tested whether Vls and Tud can assist the posterior localisation of HELICc and found that the posterior localisation of both full-length DmVas (Fig. 5C,D) and HELICc (Fig. 5G,H) were not affect in either *vls* or *tud* mutant backgrounds. Ectopic expression of SgVas^HELICc^ in *Drosophila* showed that SgVas^HELICc^ could also be posteriorly localised (Fig. 5I), and its localisation patterns resembled those of the HELICc of DmVas in various genetic backgrounds (Fig. 5J–L). These results suggest that the germ plasm localisation of HELICc was dependent on Osk but not Vls or Tud.

To identify the physical interaction between HELICc and Osk, we performed the yeast two-hybrid analysis by using DmVas as the bait and Osk or Vls as the prey. In cells singly transformed with the bait or prey plasmids, the expression of the reporter gene *lacZ* was undetectable (Fig. 5M). However, reporter signals were detected in cells co-expressing full-length DmVas and Osk or DmVas^460–621/HELICc^ and Osk (blue colour in Fig. 5N). By contrast, co-expression of the shortened HELICc (DmVas^470–661^) and Osk did not produce positive signals (upper panel of Fig. 5N). Moreover, no positive signals were detected in cells co-expressing Vls and various DmVas constructs (lower panel of Fig. 5N). This suggests that Vls does not interact with DmVas physically *in vitro*. Altogether, these results suggest that the HELICc is a target site for Osk binding and that the residues 460–469 are essential for the interaction with Osk. Nevertheless, DmVas^460–661^ containing an intact HELICc and additional 40 residues at its C-terminus produced negative results (upper panel of Fig. 5N). This coincides with a yeast two-hybrid analysis carried out by Breitwieser *et al.* (1996): a polypeptide composed of DmVas residues 446–661 failed to interact with Osk^32^. In order to verify whether the 40 residues also interfere with the Osk–HELICc interaction in *Drosophila*, we preformed co-immunoprecipitation experiments. Our results showed that both DmVas^460–621/HELICc^ and DmVas^460–661^ could be co-precipitated with Osk in the ovary extract (Supplementary Fig. S2), suggesting that the 40 residues C-terminal to HELICc hindered the Osk–HELICc interaction in the yeast cells but not in the *Drosophila* oocytes. To provide further *in vivo* evidence for the interaction between HELICc and Osk, we expressed the *UAS*–*osk*–*bcd* 3′ UTR transgene in the female germline by *nos*–*Gal4* for the ectopic expression of Osk in the oocyte anterior since the 3′ untranslated region (UTR) of *bcd* mRNA brings the transcript to the anterior cortex^29^,^40^. As a control, we observed the posterior colocalisation of DmVas^460–621/HELICc^ with Osk (Fig. 5O–Q). In the oocytes co-expressing *osk–bcd* 3′ UTR and *Dmvas*^460–621*/HELICc*^ transgenes, we also detected the colocalisation of DmVas^460–621/HELICc^ with Osk in the anterior cortex as anticipated (Fig. 5R–T).

### Gln527 as a key residue required for the germ plasm localisation of HELICc

**Figure 6:**
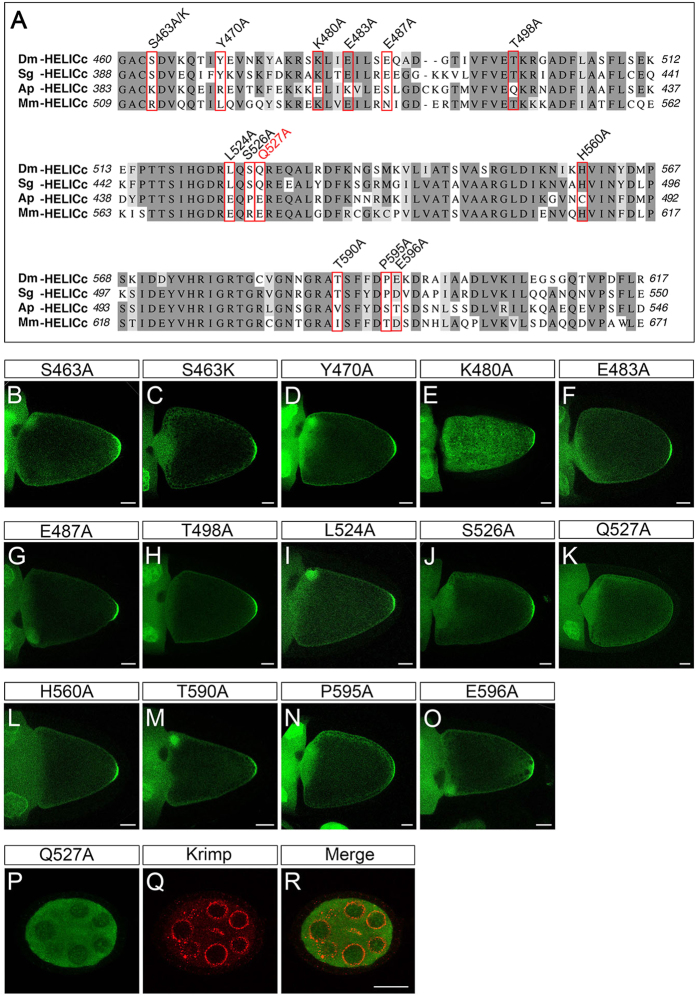
Identification of amino acid residues essential to the posterior localisation of *Drosophila* Vasa (DmVas) in the helicase superfamily C-terminal domain (HELICc). (**A**) Multiple sequence alignment of HELICc domains belonging to Vas orthologs of *Drosophila melanogaster* (Dm), the grasshopper *Schistocerca gregaria* (Sg), the pea aphid *Acyrthosiphon pisum* (Ap), and the mouse *Mus musculus* (Mm). Dark grey: conserved residues, Light grey: residues with similar properties. Residues substituted by Ala or Lys for the localisation assays shown in panels (**B**–**O**) are highlighted with red boxes. (**B–O**) Localisation analysis of green fluorescent protein (GFP)-tagged DmVas^460–661^ proteins with replaced amino acid residues in the HELICc sequence. Stage-10 egg chambers were stained using the anti-GFP antibody (green). Anterior is to the left and posterior is to the right. Scale bars, 25 μm. (**B**) DmVas^460–661/S463A^: replacement of the Ser463 with Ala is designated as S463A, and this applies to the other replacements. (**C**) DmVas^460–661/S463K^. (**D**) DmVas^460–661/Y470A^. (**E**) DmVas^460–661/K480A^. (**F**) DmVas^460–661/483A^. (**G**) DmVas^460–661/E487A^. (**H**) DmVas^460–661/T498A^. (**I**) DmVas^460–661/L524A^. (**J**) DmVas^460–661/S526A^. (**K**) DmVas^460–661/Q527A^. (**L**) DmVas^460–661/H560A^. (**M**) DmVas^460–661/T590A^. (**N**) DmVas^460–661/P595A^. (**O**) DmVas^460–661/E596A^. All of the previously described DmVas^460–661^ variants could be localised to the germ plasm, except DmVas^460–661/Q527A^, shown in panel (**K**). (**P–R**) Stage-5 egg chambers expressing GFP-DmVas^460–661/Q527A^ were double stained using the anti-GFP (green) and anti-Krimp antibodies (red). (**P**) GFP-DmVas^460–661/Q527A^ was not colocalised with (**Q**) Krimp in the nurse cells. Anterior is to the left and posterior is to the right. Scale bars, 20 *μ*m.

Posterior localisation of SgVas^HELICc^ to the germ plasm in *Drosophila* (Fig. 3H–H”’) implies that the HELICc domains of SgVas and DmVas share common amino acids for interacting with Osk. To identify the amino acids critical for the germ plasm localisation of HELICc, we thus aligned the sequences from HELICc domains of DmVas, SgVas, and ApVas1 and selected 13 residues conserved in both DmVas and SgVas but not in ApVas1 (Fig. 6A). These include Ser463, an amino acid located within the first 10 amino acids (residues 460–469) of HELICc that have been demonstrated essential for germ plasm/nuage localisation and Osk interaction (Figs 3D–D”’,4D–D” and 5N). We then successively replaced all of the 13 target residues with Ala and monitored the localisation pattern of each HELICc with a single amino acid substitution. Our results showed that replacement of the Gln527 with Ala (Q527A) abolished the posterior localisation of HELICc (Fig. 6K). By contrast, restriction of HELICc to the germ plasm could still be visualized in the remaining substitutions including S463A (Fig. 6B,D–J,L–O). Further replacement of serine at position 463 with the basic amino acid lysine (S463K) did not impair HELICc localisation (Fig. 6C), indicating that Ser463 is not a key residue for germ plasm localisation of HELICc. In addition to germ plasm localisation, Q527A substitution abolished the nuage localisation of HELICc (Fig. 6P–R). To exclude the possibility that the absence of the posterior localisation of DmVas^460–661/Q527A^ was caused by its weak expression or absence of expression, we compared the expression of DmVas^460–661^ and DmVas^460–661/Q527A^ by performing western blot analysis and found that both proteins had similar expression levels in the ovary (Supplementary Fig. S3).

### N-terminal sequence of DmVas is required for abdominal segment and pole cell formation during embryogenesis

**Figure 7:**
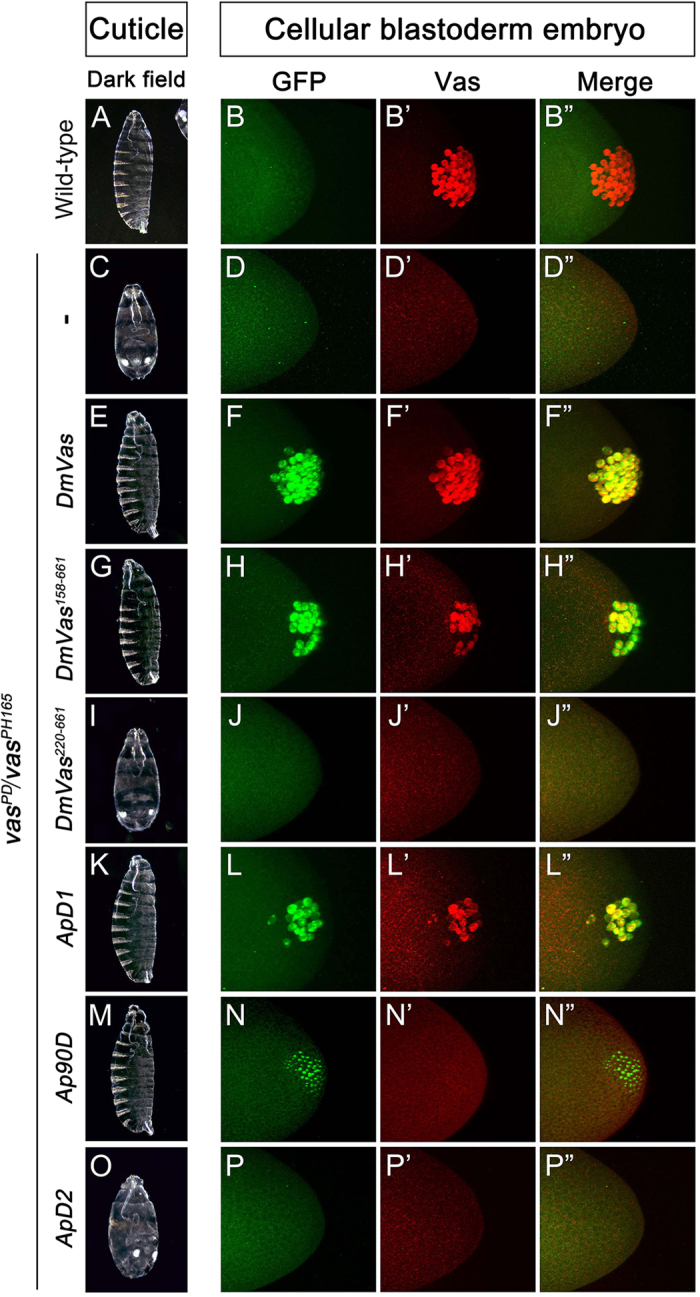
Rescue *vas* mutant defects by *Drosophila* Vasa (DmVas) variants. Examination of abdominal segments and pole cells in embryos of wild-type or *vas* mutants expressing truncated or chimeric DmVas proteins. Genotypes: (**A**–**B”**) Wild-type: Oregon-R; (**C**–**D”**) *vas* mutant: *vas*^*PD*^*/vas*^*PH165*^; (**E**–**F”**) *vas* mutants expressing GFP-DmVas, (**G**–**H”**) GFP-DmVas^158–661^, (**I**–**J”**) GFP-DmVas^220–661^, (**K**–**L”**) GFP-ApD1 (ApVas1^1–60^ + DmVas^158–661^), (**M**–**N”**) GFP-Ap90D (ApVas1^1–90^ + DmVas^158–661^), and (**O**–**P”**) GFP-ApD2 (ApVas1^1–135^ + DmVas^220–661^). (**A**,**C**,**E**,**G**,**I**,**K**,**M**,**O**) Cuticle preparations. Anterior is at the top. (**A**) Wild-type. (**C**) *vas*^*PD*^*/vas*^*PH165*^ embryo: no abdomen. (**E**,**G**,**K**,**M**) GFP-DmVas, GFP-DmVas^158–661^, GFP-ApD1, and GFP-Ap90D rescued abdominal defect. (**I**,**O**) GFP-DmVas^220–661^ and GFP-ApD2 did not rescue abdomen formation. (**B–B”**,**D–D”**,**F–F”**,**H–H”**,**J–J”**,**L–L”**,**N–N”**,**P–P’**) Z-stacks of confocal microscopic images of cellular blastoderm embryos double stained with anti-GFP and anti-Vas antibodies to visualise GFP-Vas variants (green) and endogenous Vas (red), respectively. Posterior is to the right. (**B**–**B”**) Wild-type. (**D**–**D”**) *vas*^*PD*^*/vas*^*PH165*^ embryo: no pole cell. (**F**–**F”**) GFP-DmVas rescued pole cell formation. (**H**–**H”**,**L**–**L”**) GFP-DmVas^158–661^ and GFP-ApD1 partially restored the pole cell number. (**J**–**J”**,**P**–**P”**) GFP-DmVas^220–661^ and GFP-ApD2 could not rescue pole cell formation. (**N**–**N”**) GFP-Ap90D accumulated in the posterior pole, but no pole cell was identified in most embryos examined.

To explore the functions of the DmVas sequences N-terminal to the helicase core domain, we introduced transgenes encoding truncated DmVas proteins with various lengths of N-termini into the *vas* mutant (*vas*^*PD*^*/vas*^*PH165*^)^7^,^9^ and examined the recovered phenotypes. Compared with the wild-type embryos (Fig. 7A,B–B”), no abdominal segments or pole cells could be identified in the *vas* mutant embryos (Fig. 7C,D–D”). Expression of full-length DmVas in *vas* mutants recovered eight abdominal segments and 34.4 pole cells on average (Fig. 7E,F–F”; Supplementary Table S1), resembling the phenotypes observed in the wild-type embryos (Fig. 7A,B–B”). In the *vas* mutant embryos expressing DmVas^158–661^, a DmVas protein without the first 157 amino acids, we found that all the eight abdominal segments were formed (Fig. 7G; Supplementary Table S1) but only 20.6 pole cells, on average, were recovered (Fig. 7H–H”; Supplementary Table S1). However, when all the 219 amino acids N-terminal to the DEXDc domain were deleted (DmVas^220–661^; Fig. 7I,J–J”), the phenotype resembled that of the *vas* mutant (Fig. 7C,D–D”). We further analysed the ApVas1 N-terminal sequence to understand whether it could substitute the functions of the DmVas N-terminus. We expressed the chimeric proteins ApD1, Ap90D, or ApD2, all of whose N-termini were composed of various lengths of ApVas1 and found that the addition of the ApVas1 N-terminal sequence to DmVas^158–661^ (ApD1, Fig. 7L–L”; Ap90D, Fig. 7N–N”) or DmVas^220–661^ (ApD2, Fig. 7P–P”) did not enhance the rescue of pole cell formation defects in the *vas* mutant embryos. In comparison with ApD1, Ap90D expression (30-amino acid longer than ApD1) further hindered pole cell formation; practically, pole cells were not or barely formed (Fig. 7N–N”; Supplementary Table S1). By contrast, the expression of both ApD1 and Ap90D did not interfere with the rescue of abdominal segments (Fig. 7K,M). Nevertheless, when the sequence N-terminal to DEXDc of DmVas was replaced with the first 135 amino acids of ApVas1, its expression in the *vas* mutant embryos neither rescued the pole cells nor the abdominal segments (ApD2, Fig. 7O,P–P”).

## Discussion

In this study, we began by expressing ApVas1 in *Drosophila* female germline and found that ApVas1, unlike the endogenous Vas protein (DmVas), was not localised to the germ plasm of oocyte (Fig. 1). This suggests that the molecular machinery for anchoring Vas to the germ plasm is not conserved between the pea aphid and *Drosophila*. Because a pea aphid homolog of *Drosophila osk* has not yet been identified^41^,^42^, we further inferred the following: (1) the pea aphid adopted an *osk*-independent machinery for assembling the germ plasm and (2) ApVas1 lacked the sequences recognised by Osk or any other factors associated with Osk. To test the aforementioned hypotheses, we performed domain swapping between ApVas1 and DmVas and identified the sequences responsible for the germ plasm localisation of DmVas. We found that the previously identified Osk-interacting motif (OIM)^31^ was not decisive to the posterior localisation of DmVas *in vivo*. Without OIM, for example, the HELICc domain alone was still localised to the germ plasm in oocytes, though localisation was postponed from stage 9 to 10 of oogenesis (Fig. 2G–H’”). Consequently, we infer that OIM can assist but not determine the interaction between DmVas and Osk.

Identification of HELICc as a critical domain sufficient for the germ plasm localisation of DmVas is one of our critical findings (Figs. 3B–B’”;5E). Direct interaction between HELICc and Osk, known as a key germ plasm inducer, is supported by evidence from the yeast two-hybrid assays (Fig. 5M,N), *in vivo* localisation analyses (Fig. 5R–T), and co-immunoprecipitation experiments (Supplementary Fig. S2). Recently, Jeske *et al.* (2015) presented crystal structure of the N-terminal LOTUS domain of Osk and, via GST-pull down assay, demonstrated that it could interact with the helicase core of DmVas, a polypeptide region containing both DEXDc and HELICc^43^. This result, together with our findings, strongly suggests that HELICc is a direct target of the LOTUS domain. The Q527A substitution within HELICc disabled its germ plasm localisation, moreover, further implying that Gln527 is indispensable for the Osk–Vas interaction in *Drosophil*a (Fig. 6K). Nonetheless, existing evidence does not support that Gln527 and its aligned residues in other insect species play a conserved role in the assembly of germ plasm. For example, the grasshopper *S. gregaria* has a Gln residue equivalent to Gln527 in DmVas yet a maternal germ plasm has not been identified^33^ (Supplementary Fig. S1A). The wasp *Nasonia vitripennis*, by contrast, possesses Glu rather than Gln in the HELICc of Vas that is localised to the maternal germ plasm^44^ (Supplementary Fig. S1B). Accordingly, we suggest that Gln527 responsible for the Osk–Vas interaction may only be conserved in *Drosophila* and its closely related genera for constructing a unique ‘Vas networking’ involved in the formation of germ plasm.

**Figure 8:**
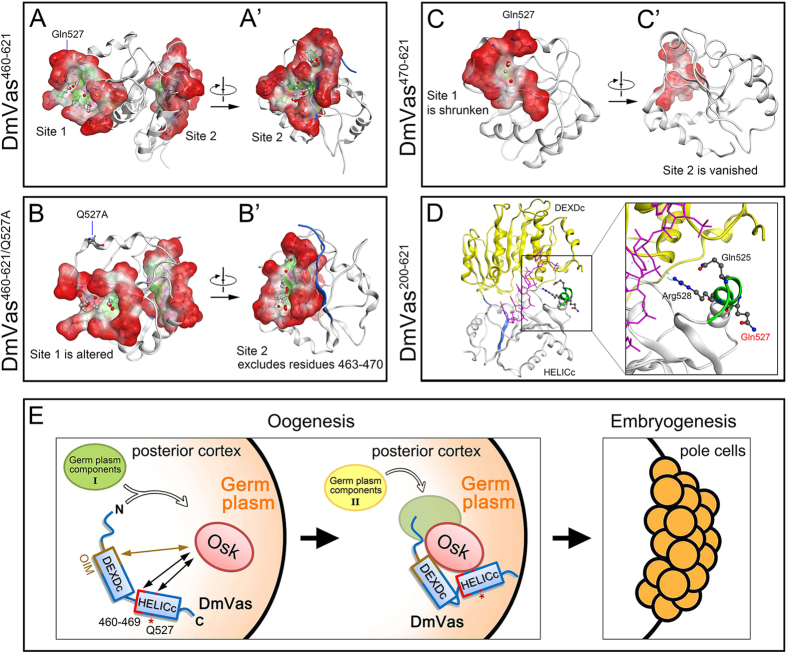
Structures and predicted protein interaction sites in DmVas^460–621^, DmVas^460–621/Q527A^, and DmVas^470–621^. (**A–C**) Molecular dynamics (MD) simulated structures of DmVas^460–621^, DmVas^460–621/Q527A^, and DmVas^470–621^ after 100-ns simulations (Supplementary Methods). Protein–protein interaction sites were predicted using the Site Finder of the Molecular Operating Environment (MOE) software package, and dummy atoms were placed within the MD-simulated structures. Red and grey dummy atoms represent potential hydrophilic and hydrophobic interactions, respectively. Residues located in the individual interaction sites are listed in the supplementary material Table S2. Red: exposed surface; pink: hydrophilic region; green: hydrophobic region; blue: the back bone of residues 460–469. Panels (**A’**–**C’**) are horizontal rotations of (**A**–**C**), respectively. (**A**,**A’**) DmVas^460–621^: Gln527 is located within Site 1; 6 residues (Ser463, Asp464, Val465, Lys466, Gln467, and Thr468) from amino acids 460–469 are located within Site 2. (**B**,**B’**) DmVas^460–621/Q527A^: a conformational change excluded the residues around Q527A from Site 1 and residues 460–469 from Site 2. (**C**,**C’**) DmVas^470–621^: Site 1 was greatly reduced in size; Site 2 vanished. (**D**) Crystal structure of DmVas bound with the poly(U) RNA (PDB code: 2DB3; residues 200-621). Residues Gln525 and Arg528 interacted with the RNA, whereas Gln527 did not. Yellow: DEXDc; white: HELICc; pink: poly(U) RNA; blue: residues 460–469; green: QxxR RNA-binding motif. (**E**) A presumptive model for the germ plasm localisation of DmVas. After the Oskar (Osk) protein accumulated in the posterior pole of the oocyte at Stage 9 of oogenesis, it interacted with DmVas through residues Gln527 (Q527) and 460–469 in HELICc. The OIM (residues 163–319) might promote or stabilise the interaction between HELICc and Osk. Moreover, we proposed that some germ plasm components (I) could pre-associate with the DmVas during its transportation from nurse cells to the oocyte. In the germ plasm, DmVas could localise additional germ plasm components (II) with the aid of Osk and/or other Osk-bound molecules. Pole cell formation follows germ plasm assembly in the early embryogenesis.

To understand how Gln527 participated in the Osk–Vas interaction in *Drosophila*, we performed MD simulations and protein-protein interaction site prediction to analyse the possible conformational change in HELICc caused by the Q527A substitution. In the MD simulated structure of HELICc, two protein–protein interaction pockets containing the residues 460–469 and Gln527 were predicted (Fig. 8A,A’). Gln527 was identified within a predicted interaction pocket entitled as ‘site 1’ whereas residues 463–468 were included in ‘site 2’ (DmVas^460–621^ in Fig. 8A; Supplementary Table S2). The Q527A substitution excludes Arg523, Lys524 and Arg528, all of which are amino acids surrounding Gln527, from site 1. Meanwhile it expels residues 463–470 from site 2 (Fig. 8B; Supplementary Table S2). Consequently, conformational distortion of sites 1 and 2 in HELICc may explain why DmVas^460–661/Q527A^ cannot be localised to the germ plasm. According to the published crystal structure of DmVas, the residue Gln527 is located within the RNA-binding motif QxxR^22^. However, because the side chain of Gln527 flips outside the RNA-binding pocket, we believe that it does not directly interact with the target RNA (Fig. 8D). Further evidence is required for understanding whether the protruding side chain of Gln527 is a direct target of Osk. We also simulated the structure of DmVas^470–621^ and found that truncation of the HELICc N-terminal sequence, namely the residues 460–469 of DmVas, led to distortion and shrinkage of site 1 and a deletion of site 2 (Fig. 8C,C’). The fact that DmVas^470–661^ could not be detected in the posterior germ plasm (Fig. 3D–D”’) suggests that the residues 460–469, similar to Gln527, contribute to the Osk–Vas interaction, and that the predicted sites 1 and 2 could play a role. Liang *et al.* (1994) identified four EMS-induced mutations in HELICc that could disrupt germ plasm localisation of Vas^23^. Likewise, a recent study carried out by Dehghani and Lasko (2015) shows that substitution of Thr546 with Ala (T546A) in HELICc results in the same outcome^45^. MD simulation shows that they are respectively located within site 1 (Ser518, His520, Thr546) and site 2 (Val465, Gly587). MD simulation predicts that: (1) these 5 amino acids are respectively located within site 1 (Ser518, His520, Thr546) and site 2 (Val465, Gly587); (2) the Q527A substitution expels Thr546 from site 1 and Val465 from site 2; and (3) the deletion of residues 460–469 excludes His520/Thr546 from site 1 (Supplementary Table S2). We therefore infer that Gln527 and residues 460–469 stabilize the conformation of HELICc.

In addition to germ plasm localisation, Gln527 and the residues 460–469 were involved in the nuage localisation of DmVas because the Q527A substitution and the truncation of residues 460–469 disabled the nuage localisation of HELICc (Fig. 4D–D””). However, unlike to the germ plasm, Osk protein is not translated in the nurse cells and the restriction of DmVas to the nuage is unaffected in the *osk* mutant background^46^,^47^. We thus infer that HELICc is not only essential for ‘Osk-dependent’ germ plasm localisation but also for ‘Osk-independent’ nuage localisation. Within the nucleus of nurse cell, nascent Piwi-interacting RNA precursors that are tethered to the nuclear DEAD-box protein UAP56 penetrate through the nuclear pore, thus bridging the connection with DmVas in the perinuclear nuage^48^. This suggests that the nuage localisation of DmVas is more likely mediated by an RNA–protein, rather than a protein–protein interaction. Moreover, given that side chains of Gln527 and the residues 460–469 are neutral or negatively charged (except the positively charged Lys466; see Supplementary Fig. S1A), they are unlikely to bind to the negatively charged phosphate groups on the Piwi-interacting RNA backbones. Thus, Gln527 and the residues 460–469 may only contribute to the conformational integrity of HELICc for the nuage localisation of DmVas.

Regarding the N-terminal region of DmVas, we found that residues 1–157 and 158–219 play distinct roles. In the *vas* mutants, deletion of the first 157 amino acids led to a rescue of all abdominal segments but only recovered part of the pole cells (Fig. 7G,H–H”; Supplementary Table S1). However, further deletion of the residues 158–219 did not rescue any of the abdominal segments or pole cells (Fig. 7I,J–J”). These results suggest that the residues 1–157 are exclusively required for pole cell formation, whilst the residues 158–219 are important to the development of pole cells and abdominal segments. Although the N-termini of both DmVas and ApVas1 have the RGG RNA-binding motifs in common, compensation of the truncated N-terminus in DmVas with corresponding region in ApVas1 could neither increase the number of pole cells nor rescue any abdominal segments (Fig. 7 and Supplementary Table S1). This implies that the divergent sequences in the N-termini of ApVas1 and DmVas, although both of which contain RGG repeats, are not functionally exchangeable between *Drosophila* and the pea aphid.

In conclusion, molecular dissection of DmVas has unveiled the relationship between sequence and functions of this versatile germline marker in *Drosophila* (Fig. 8E). The HELICc is sufficient for the nuage and germ plasm localisation of DmVas, whereas the N-terminal divergent sequence of DmVas is critical for pole cell and abdomen formation. Nonetheless, we find that several questions remain unanswered. These include the following: (1) Why is the nuage localisation of DmVas prerequisite for its germ plasm localisation? (2) Is any modification of DmVas required before its recruitment to the germ plasm? If so, does this take place in the nuage? (3) Which germline components are associated with DmVas in the nuage, with the transporting DmVas, and later with the germ plasm-localised DmVas? In addition to those of DmVas, we anticipate that unveiling the versatile functions of *vas* genes in other insects will enlighten the ancestral and diverse roles of *vas* in germline specification as well as early embryonic development.

## Methods

### *Drosophila* stocks and transgenes

Oregon R was used as the wild-type strain. The *w*^1118^ strain was used as a host for P-element-mediated transgenesis. Fly stocks were raised at 25 °C on a standard cornmeal medium. The mutant alleles used were as follows: *osk*^54 12^, *vas*^*PD* 7^, *vas*^*PH165 9*^, *vls*^*null* 38^, *tud*^*tux*46 39^, P{*UASp-osk-bcd 3′UTR*}^40^, P{*UASp-osk*}^49^. All the other stocks used were provided by the Bloomington Stock Centre. The following transgenic stocks were generated in this study: P{*gfp-Apvas1*}, P{*gfp-Dmvas*}, P{*gfp-ApD1*}, P{*gfp-ApD2*}, P{*gfp-ApD3*}, P{*gfp-DAp1*}, P{*gfp-DAp2*}, P{*gfp-DmVas*^*1−*320^}, P{*gfp-DmVas*^158−661^}, P{*gfp-DmVas*^220−661^}, P{*gfp-DmVas*^321–661^}, P{*gfp-DmVas*^1–460^}, P{*gfp-DmVas*^460–661^}, P{*gfp-DmVas*^460−621*/HELICc*^}, P{*gfp-DmVas*^470–661^}, P{*gfp-ApD*^*HELICc*^}, P{*gfp-ApVas*^*HELICc*^}, P{*gfp-SgVas*^*HELICc*^}, P{*gfp-mvh*^*HELICc*^}, P{*gfp-DmVas*^*460−661/S463A*^}, P{*gfp-DmVas*^*460−661/S463K*^}, P{*gfp-DmVas*^*460−661/Y470A*^}, P{*gfp-DmVas*^*460−661/K480A*^}, P{*gfp-DmVas*^*460−661/E483A*^}, P{*gfp-DmVas*^*460−661/E487A*^}, P{*gfp-DmVas*^*460−661/T498A*^}, P{*gfp-DmVas*^*460−661/L524A*^}, P{*gfp-DmVas*^*460−661/S526A*^}, P{*gfp-DmVas*^*460−661/Q527A*^}, P{*gfp-DmVas*^*460−661/H560A*^}, P{*gfp-DmVas*^*460−661/T590A*^}, P{*gfp-DmVas*^*460−661/P595A*^}, and P{*gfp-DmVas*^*460−661/E596A*^}. All of these transgene constructs were cloned into the p{*Pmat-tub67c*:*gfp*} vector containing maternal *tubulin 67c* promoter for female germline expression^26^. Each of the fragments of *Drosophila vas* coding sequence (CDS) were PCR amplified from pP*vas:egfp-vas*^27^. The CDSs encoding the HELICc of aphid and grasshopper *vas* were RT-PCR amplified from ovarian lysates. The CDS encoding the HELICc of *mvh* was obtained through PCR amplification of the testis cDNA (a gift from Dr. Yung-Hao Ching). The QuikChange Lightning Site-Directed Mutagenesis kit (Agilent Technologies) was used to generate amino acid substitutions in the HELICc of DmVas.

### Ovary and embryo whole mount immunostaining

The ovary and embryo immunostaining protocols used was previously described^50^,^51^. The primary antibodies used were as follows: mouse anti-GFP antibody (1:100; Roche), rabbit anti-Osk antibody (1:100; a gift from Dr. Tze-Bin Chou), rabbit anti-Tudor antibody (1:100; a gift from Dr. Akira Nakamura), rabbit anti-Krimp antibody (1:500; a gift from Dr. Toshie Kai) and rat anti-Vas antibody (1:100; Developmental Studies Hybridoma Bank). The fluorescently labelled secondary antibodies used were as follows: goat anti-mouse Alexa Fluor 488 (1:100; Invitrogen), goat anti-rabbit Alexa Fluor 647 (1:100; Invitrogen) and goat anti-rat Alexa Fluor 647 (1:100; Invitrogen).

### Yeast two-hybrid assay

The yeast strain Y187 was used. Yeast culturing and the colony lift assay were performed according to the Clontech yeast two-hybrid handbook. Yeast transformation was performed using the Frozen-EZ Yeast Transformation II kit (Zymo Research). The full-length DmVas CDS (1–661) and other truncated fragments (460–661, 460–620, and 470–661) were PCR amplified and subcloned into pAS2-1 (Clontech) for generating GAL4 DNA-binding domain fusion proteins. The full-length Osk and Vls CDSs were PCR amplified and subcloned into pACT2 (Clontech) for generating GAL4 activation domain fusion proteins. The *osk* CDS was PCR amplified from the EST clone LD24944. The *vls* CDS was obtained through RT-PCR amplification of the RNA extracted from the *Drosophila* ovary.

## Additional Information

**How to cite this article**: Wang, S.-C. *et al.* Germ plasm localisation of the HELICc of Vasa in *Drosophila*: analysis of domain sufficiency and amino acids critical for localisation. *Sci. Rep.* **5**, 14703; doi: 10.1038/srep14703 (2015).

## Supplementary Material

Supplementary Information

## References

[b1] Extavour C. G. & Akam M. Mechanisms of germ cell specification across the metazoans: epigenesis and preformation. Development 130, 5869–5884 (2003). 14597570 10.1242/dev.00804

[b2] Hay B., Ackerman L., Barbel S., Jan L. Y. & Jan Y. N. Identification of a component of *Drosophila* polar granules. Development 103, 625–640 (1988). 3150351 10.1242/dev.103.4.625

[b3] Schupbach T. & Wieschaus E. Maternal-effect mutations altering the anterior-posterior pattern of the *Drosophila* embryo. Roux. Arch. Dev. Biol. 195, 302–317 (1986). 10.1007/BF00376063 28306055

[b4] Lasko P. F. & Ashburner M. The product of the *Drosophila* gene *vasa* is very similar to eukaryotic initiation factor-4A. Nature 335, 611–617 (1988). 3140040 10.1038/335611a0

[b5] Raz E. The function and regulation of *vasa*-like genes in germ-cell development. Genome Biol 1, REVIEWS1017.1-1017.5 (2000). 10.1186/gb-2000-1-3-reviews1017 PMC138859 11178242

[b6] Lasko P. The DEAD-box helicase Vasa: evidence for a multiplicity of functions in RNA processes and developmental biology. Biochim. Biophys. Acta. 1829, 810–816 (2013). 23587717 10.1016/j.bbagrm.2013.04.005

[b7] Hay B., Jan L. Y. & Jan Y. N. Localization of vasa, a component of *Drosophila* polar granules, in maternal-effect mutants that alter embryonic anteroposterior polarity. Development 109, 425–433 (1990). 2119289 10.1242/dev.109.2.425

[b8] Lasko P. F. & Ashburner M. Posterior localization of *vasa* protein correlates with, but is not sufficient for, pole cell development. Genes Dev. 4, 905–921 (1990). 2384213 10.1101/gad.4.6.905

[b9] Styhler S., Nakamura A., Swan A., Suter B. & Lasko P. *vasa* is required for GURKEN accumulation in the oocyte, and is involved in oocyte differentiation and germline cyst development. Development 125, 1569–1578 (1998). 9521895 10.1242/dev.125.9.1569

[b10] Dahanukar A. & Wharton R. P. The Nanos gradient in *Drosophila* embryos is generated by translational regulation. Genes Dev. 10, 2610–2620 (1996). 8895662 10.1101/gad.10.20.2610

[b11] Gavis E. R., Lunsford L., Bergsten S. E. & Lehmann R. A conserved 90 nucleotide element mediates translational repression of *nanos* RNA. Development 122, 2791–2800 (1996). 8787753 10.1242/dev.122.9.2791

[b12] Markussen F. H., Michon A. M., Breitwieser W. & Ephrussi A. Translational control of *oskar* generates short OSK, the isoform that induces pole plasma assembly. Development 121, 3723–3732 (1995). 8582284 10.1242/dev.121.11.3723

[b13] Rongo C., Gavis E. R. & Lehmann R. Localization of *oskar* RNA regulates *oskar* translation and requires Oskar protein. Development 121, 2737–2746 (1995). 7555702 10.1242/dev.121.9.2737

[b14] Ghabrial A. & Schupbach T. Activation of a meiotic checkpoint regulates translation of Gurken during *Drosophila* oogenesis. Nat. Cell Biol. 1, 354–357 (1999). 10559962 10.1038/14046

[b15] Johnstone O. & Lasko P. Interaction with eIF5B is essential for Vasa function during development. Development 131, 4167–4178 (2004). 15280213 10.1242/dev.01286

[b16] Liu N., Han H. & Lasko P. Vasa promotes *Drosophila* germline stem cell differentiation by activating mei-P26 translation by directly interacting with a (U)-rich motif in its 3′ UTR. Genes Dev. 23, 2742–2752 (2009). 19952109 10.1101/gad.1820709 PMC2788330

[b17] Kiledjian M. & Dreyfuss G. Primary structure and binding activity of the hnRNP U protein: binding RNA through RGG box. EMBO. J. 11, 2655–2664 (1992). 1628625 10.1002/j.1460-2075.1992.tb05331.x PMC556741

[b18] McBride A. E. & Silver P. A. State of the arg: protein methylation at arginine comes of age. Cell 106, 5–8 (2001). 11461695 10.1016/s0092-8674(01)00423-8

[b19] Styhler S., Nakamura A. & Lasko P. VASA localization requires the SPRY-domain and SOCS-box containing protein, GUSTAVUS. Dev. Cell 3, 865–876 (2002). 12479811 10.1016/s1534-5807(02)00361-1

[b20] Kugler J. M., Woo J. S., Oh B. H. & Lasko P. Regulation of *Drosophila vasa in vivo* through paralogous cullin-RING E3 ligase specificity receptors. Mol. Cell Biol. 30, 1769–1782 (2010). 20123973 10.1128/MCB.01100-09 PMC2838069

[b21] Bork P. & Koonin E. V. An expanding family of helicases within the ‘DEAD/H’ superfamily. Nucleic Acids Res. 21, 751–752 (1993). 8382805 10.1093/nar/21.3.751 PMC309186

[b22] Sengoku T., Nureki O., Nakamura A., Kobayashi S. & Yokoyama S. Structural basis for RNA unwinding by the DEAD-box protein *Drosophila* Vasa. Cell 125, 287–300 (2006). 16630817 10.1016/j.cell.2006.01.054

[b23] Liang L., Diehl-Jones W. & Lasko P. Localization of vasa protein to the *Drosophila* pole plasm is independent of its RNA-binding and helicase activities. Development 120, 1201–1211 (1994). 8026330 10.1242/dev.120.5.1201

[b24] Chang C.-c. *et al.* *Apvasa* marks germ-cell migration in the parthenogenetic pea aphid *Acyrthosiphon pisum* (Hemiptera: Aphidoidea). Dev. Genes Evol. 217, 275–287 (2007). 17333259 10.1007/s00427-007-0142-7

[b25] Lin G. W., Cook C. E., Miura T. & Chang C.-c. Posterior localization of ApVas1 positions the preformed germ plasm in the sexual oviparous pea aphid *Acyrthosiphon pisum*. EvoDevo 5, 18 (2014). 24855557 10.1186/2041-9139-5-18 PMC4030528

[b26] Schuldt A. J. *et al.* Miranda mediates asymmetric protein and RNA localization in the developing nervous system. Genes Dev. 12, 1847–1857 (1998). 9637686 10.1101/gad.12.12.1847 PMC316902

[b27] Sano H., Nakamura A. & Kobayashi S. Identification of a transcriptional regulatory region for germline-specific expression of *vasa* gene in *Drosophila melanogaster*. Mech. Develop. 112, 129–139 (2002). 10.1016/s0925-4773(01)00654-2 11850184

[b28] Kim-Ha J., Smith J. L. & Macdonald P. M. *Oskar* mRNA is localized to the posterior pole of the *Drosophila* oocyte. Cell 66, 23–35 (1991). 2070416 10.1016/0092-8674(91)90136-m

[b29] Ephrussi A. & Lehmann R. Induction of germ cell formation by *oskar*. Nature 358, 387–392 (1992). 1641021 10.1038/358387a0

[b30] Ephrussi A., Dickinson L. K. & Lehmann R. Oskar organizes the germ plasm and directs localization of the posterior determinant nanos. Cell 66, 37–50 (1991). 2070417 10.1016/0092-8674(91)90137-n

[b31] Anne J. Targeting and anchoring Tudor in the pole plasm of the *Drosophila* oocyte. PLoS One 5, e14362 (2010). 21179512 10.1371/journal.pone.0014362 PMC3002268

[b32] Breitwieser W., Markussen F. H., Horstmann H. & Ephrussi A. Oskar protein interaction with Vasa represents an essential step in polar granule assembly. Genes Dev. 10, 2179–2188 (1996). 8804312 10.1101/gad.10.17.2179

[b33] Chang C.-c., Dearden P. & Akam M. Germ line development in the grasshopper *Schistocerca gregaria*: *vasa* as a marker. Dev. Biol. 252, 100–118 (2002). 12453463 10.1006/dbio.2002.0840

[b34] Hay B., Jan L. Y. & Jan Y. N. A protein component of *Drosophila* polar granules is encoded by *vasa* and has extensive sequence similarity to ATP-dependent helicases. Cell 55, 577–587 (1988). 3052853 10.1016/0092-8674(88)90216-4

[b35] Findley S. D., Tamanaha M., Clegg N. J. & Ruohola-Baker H. Maelstrom, a *Drosophila* spindle-class gene, encodes a protein that colocalizes with Vasa and RDE1/AGO1 homolog, Aubergine, in nuage. Development 130, 859–871 (2003). 12538514 10.1242/dev.00310

[b36] Lim A. K. & Kai T. Unique germ-line organelle, nuage, functions to repress selfish genetic elements in *Drosophila melanogaster*. Proc. Natl. Acad. Sci. USA 104, 6714–6719 (2007). 17428915 10.1073/pnas.0701920104 PMC1871851

[b37] Lehmann R. & Nusslein-Volhard C. Abdominal segmentation, pole cell formation, and embryonic polarity require the localized activity of *oskar*, a maternal gene in *Drosophila*. Cell 47, 141–152 (1986). 3093084 10.1016/0092-8674(86)90375-2

[b38] Cavey M., Hijal S., Zhang X. & Suter B. *Drosophila valois* encodes a divergent WD protein that is required for Vasa localization and Oskar protein accumulation. Development 132, 459–468 (2005). 15634703 10.1242/dev.01590

[b39] Thomson T. & Lasko P. *Drosophila tudor* is essential for polar granule assembly and pole cell specification, but not for posterior patterning. Genesis 40, 164–170 (2004). 15495201 10.1002/gene.20079

[b40] Tanaka T. & Nakamura A. The endocytic pathway acts downstream of Oskar in *Drosophila* germ plasm assembly. Development 135, 1107–1117 (2008). 18272590 10.1242/dev.017293

[b41] The International Aphid Genomics Consortium Genome sequence of the pea aphid *Acyrthosiphon pisum*. PLoS Biol. 8, e1000313 (2010). 20186266 10.1371/journal.pbio.1000313 PMC2826372

[b42] Legeai F. *et al.* AphidBase: a centralized bioinformatic resource for annotation of the pea aphid genome. Insect Mol. Biol. 19 Suppl 2, 5–12 (2010). 20482635 10.1111/j.1365-2583.2009.00930.x PMC4372297

[b43] Jeske M. *et al.* The crystal structure of the *Drosophila* germline inducer Oskar identifies two domains with distinct Vasa helicase- and RNA-binding activities. Cell Rep. 12, 587–598 (2015). 26190108 10.1016/j.celrep.2015.06.055

[b44] Lynch J. A. *et al.* The phylogenetic origin of *oskar* coincided with the origin of maternally provisioned germ plasm and pole cells at the base of the Holometabola. PLoS Genet. 7, e1002029 (2011). 21552321 10.1371/journal.pgen.1002029 PMC3084197

[b45] Dehghani M. & Lasko P. *In vivo* mapping of the functional regions of the DEAD-box helicase Vasa. Biol. Open 4, 450–462 (2015). 25795910 10.1242/bio.201410579 PMC4400588

[b46] Webster P. J., Liang L., Berg C. A., Lasko P. & Macdonald P. M. Translational repressor *bruno* plays multiple roles in development and is widely conserved. Genes Dev. 11, 2510–2521 (1997). 9334316 10.1101/gad.11.19.2510 PMC316560

[b47] Kim-Ha J., Kerr K. & Macdonald P. M. Translational regulation of *oskar* mRNA by Bruno, an ovarian RNA-binding protein, is essential. Cell 81, 403–412 (1995). 7736592 10.1016/0092-8674(95)90393-3

[b48] Zhang F. *et al.* UAP56 couples piRNA clusters to the perinuclear transposon silencing machinery. Cell 151, 871–884 (2012). 23141543 10.1016/j.cell.2012.09.040 PMC3499805

[b49] Zimyanin V., Lowe N. & St Johnston D. An *oskar*-dependent positive feedback loop maintains the polarity of the *Drosophila* oocyte. Curr. Biol. 17, 353–359 (2007). 17275299 10.1016/j.cub.2006.12.044 PMC1885951

[b50] Lin M. D. *et al.* *Drosophila* processing bodies in oogenesis. Dev. Biol. 322, 276–288 (2008). 18708044 10.1016/j.ydbio.2008.07.033

[b51] Lin M. D. *et al.* Expression of phosphatase of regenerating liver family genes during embryogenesis: an evolutionary developmental analysis among *Drosophila*, amphioxus, and zebrafish. BMC Dev. Biol. 13, 18 (2013). 23641863 10.1186/1471-213X-13-18 PMC3663695

