# Supplementary material for: Germ plasm localisation of the HELICc of Vasa in Drosophila: analysis of domain sufficiency and amino acids critical for localisation
Source: Sci Rep. 2015 Sep 30;5:14703. doi: 10.1038/srep14703 (PMC4588571; doi:10.1038/srep14703)
Supplement: Supplementary Information [file srep14703-s1.pdf]

Germ plasm localisation of the HELICc of Vasa in *Drosophila*: analysis of domain sufficiency and amino acids critical for localisation

Szu-Chieh Wang[1], Hao-Jen Hsu[2], Gee-way Lin[3,4], Ting-Fang Wang[1], Chun-che Chang[3,4,5]*, Ming-Der Lin[1,2]*

1. Department of Molecular Biology and Human Genetics, Tzu-Chi University, Hualien, Taiwan; 2. Department of Life Science, Tzu-Chi University, Hualien, Taiwan; 3. Laboratory for Genetics and Development, Department of Entomology/Institute of Biotechnology, College of Bioresources and Agriculture, National Taiwan University, Taipei, Taiwan; 4. Research Center for Developmental Biology and Regenerative Medicine, National Taiwan University, Taipei, Taiwan; 5. Genome and Systems Biology Degree Program, National Taiwan University, Taipei, Taiwan

*: Authors of correspondence

Chun-che Chang: chunche@ntu.edu.tw

Department of Entomology, National Taiwan University; No. 27, Lane 113, Roosevelt Road, Sec. 4, Taipei, 106, Taiwan; Tel: +886-2-3366-5578

Ming-Der Lin: mingder@mail.tcu.edu.tw

Department of Molecular Biology and Human Genetics, Tzu-Chi University; No.701, Zhongyang Rd., Sec 3. Hualien, 97004, Taiwan; Tel: +886-3-856-5301 ext. 2635

Supplementary Methods

Molecular dynamics simulations and protein-binding site prediction. The structure of the wild-type HELICc of Vasa (DmVas$^{460-621}$) was obtained from the PDB database (PDB code: 2DB3) and used as a template for the MD simulation. The HELICc was placed in a water box with dimensions 69 × 69 × 69 Å, and after energy minimisation, 28 Na^{+} and 31 Cl^{-} ions were added to neutralise the whole system in the presence of 100 mM NaCl. The patches consisted of the complex structure (1553 atoms) and 9583 SPC water molecules, including 28 Na^{+} and 31 Cl^{-} ions (30 361 atoms in total). The 10-residue truncated HELICc of DmVas (DmVas$^{460–621}$) and the Q527A-substituted HELICc of DmVas (DmVas$^{460–621/Q527A}$) systems were also constructed for comparison. All simulation systems were run for 100 ns. All MD simulations for these systems reported in the study were run with GROMACS-4.5.5, using a Gromos96 (ffG45a3) force field with an integration step size of 2 fs. The simulations were run in the *NPT* ensemble, employing the velocity-rescaling thermostat at a constant temperature of 310 K and a pressure of 1 bar. The temperature of the complex protein, lipids, and the solvent were separately coupled with a coupling time of 0.1 ps. Isotropic pressure coupling was applied with a coupling time of 0.1 ps and a compressibility of 4.5×10^{-5} bar^{-1} for the x, y, and z directions. Long-range electrostatics were calculated using the particle mesh Ewald (PME) summation algorithm with grid dimensions of 0.12 nm and an interpolation

order of 4. Lennard–Jones and short-range Coulomb interactions were cut off at 1.4 and 1.0 nm, respectively. The equilibration protocol adopted was as follows: (1) the temperature was gradually increased from 100 to 200 and 310 K. The system was run for 500 ps at each temperature. During these simulations, the complex structure remained fully restrained (k = 1000 kJ mol^{-1} nm^{-2}). (2) At 310 K, the restraints imposed on the complex structure by the force constant k were released in 3 steps, from k = 500 kJ mol^{-1} nm^{-2} to k = 250 kJ mol^{-1} nm^{-2}, and finally to k = 100 kJ mol^{-1} nm^{-2}. Each step was run for 2.0 ns. The MD simulation protocol adopted was as follows: after energy minimisation and equilibration, 100-ns production runs were performed without any restraint on the complex structure. For the protein-binding site prediction, the Site Finder tool of the Molecular Operating Environment software package (MOE; Chemical Computing Group Inc., Montreal, Canada) was used to calculate the possible active sites in the 3 simulation systems, $DmVas^{460–621}$, $DmVas^{470–621}$, and $DmVas^{460–621/Q527A}$.

Co-immunoprecipitation and Western blotting. For co-immunoprecipitation, ovaries from female flies were hand-dissected in ice-cold PBS. The ovaries were homogenized in ice-cold lysis buffer (20mM Tris-HCl pH 7.4, 150mM NaCl, 1mM EDTA, 1mM EGTA, 0.1% Triton X-100, protease inhibitors), then centrifuged at 8000 g for 10 minutes at 4ºC. The supernatant was collected and incubated with

mouse anti-GFP antibody (Roche) at 4ºC for 2 hours. The immune complex was precipitated by Protein G-conjugated magnetic beads (GE Healthcare) at 4ºC for another 3 hours. After washing with lysis buffer, bead-bound immune complex was eluted by boiling in SDS-sample buffer and followed by Western blot analysis. Western blotting was performed with standard protocol. Antibodies used in Western blot: mouse anti-GFP antibody (1:1000; Roche), mouse anti-α Tubulin antibody (1:100000; Sigma), rabbit anti-Osk antibody (1:1000; a gift from Dr. Tze-Bin Chou), rabbit anti-GFP antibody (1:2000; GeneTex). HRP conjugated goat anti-rabbit or anti-mouse secondary antibodies and were used at dilution (1:5000; GeneTex).

Supplementary Figures and legends

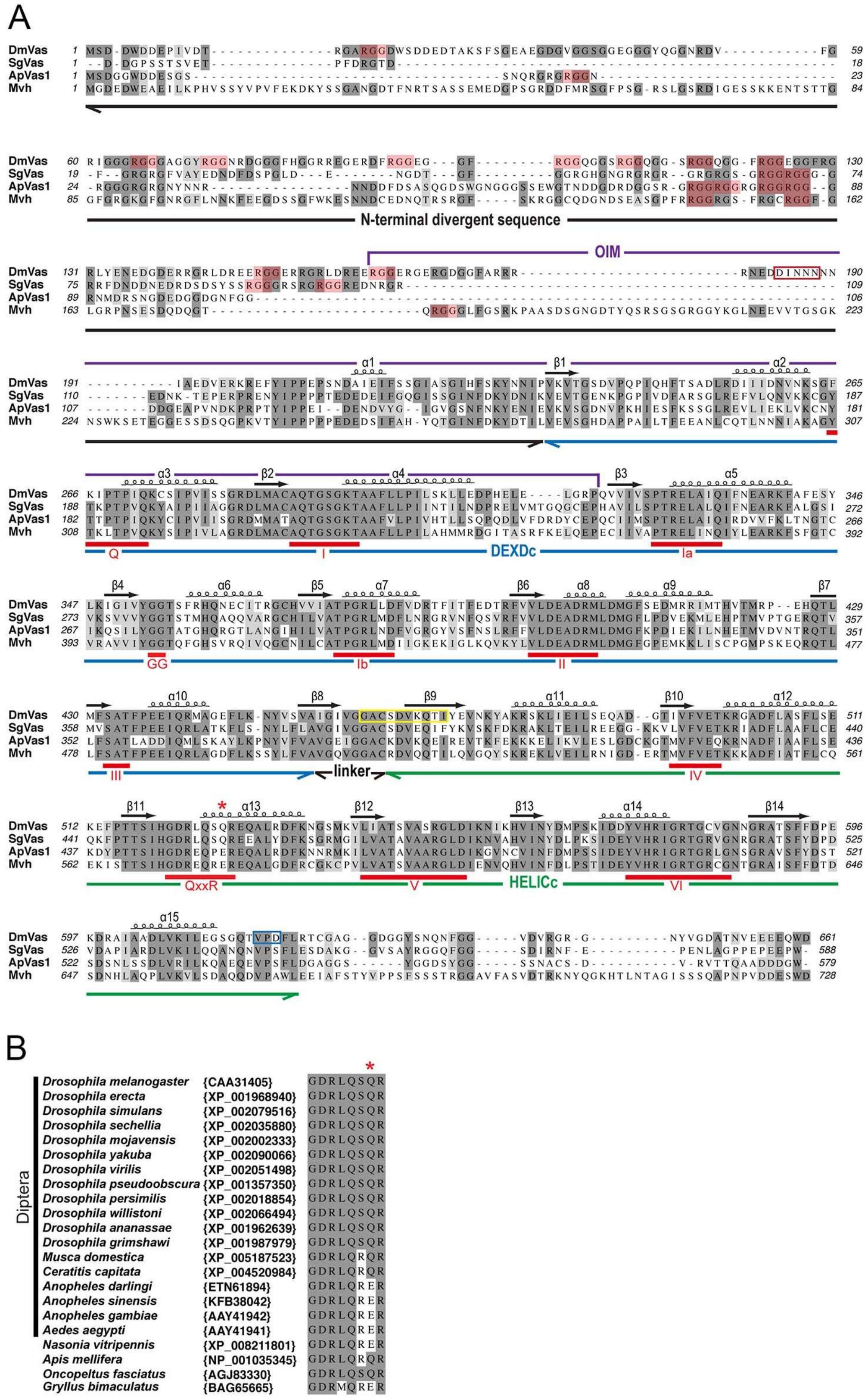

Figure S1. Sequence alignment of Vasa (Vas) orthologs from *Drosophila* and selected insect species

(**A**) Multiple sequence alignment of Vas orthologs from the fruit fly *Drosophila melanogaster* (DmVas), grasshopper *Schistocerca gregaria* (SgVas), pea aphid *Acyrthosiphon pisum* (ApVas1), and mouse *Mus musculus* (Mvh). The following features are indicated are as follows: the N-terminal divergent sequences (underlined with black bar), the RGG repeats (filled pink boxes), the DEAD-like helicases superfamily (DEXDc) domain (underlined with blue bar), the helicase superfamily C-terminal (HELICc) domain (underlined with green bar), the conserved motifs of DEAD-box helicases (underlined with red bars), and the Osk interacting motif (OIM) of DmVas (purple bars above the sequence), The DINNN motif required for Gustavus (Gus) binding (residues 184–188, red rectangle), residues 460–469 (yellow rectangle), residue Gln527 (Q527, red asterisk), and eIF5B interaction motif (residues 616–618, blue rectangle) of DmVas are also indicated. Secondary structures are marked above the sequence. Colour code for the alignment: dark grey, conserved residues; light grey, residues with similar properties. (**B**) Alignment of the RNA-binding motif QxxR of Vas orthologs from Diptera and selected insect species. Residues corresponding to Gln527 of DmVas (red asterisk) are conserved within the genus *Drosophila*. The Vas orthologs are listed by the species names, followed by the GenBank accession numbers.

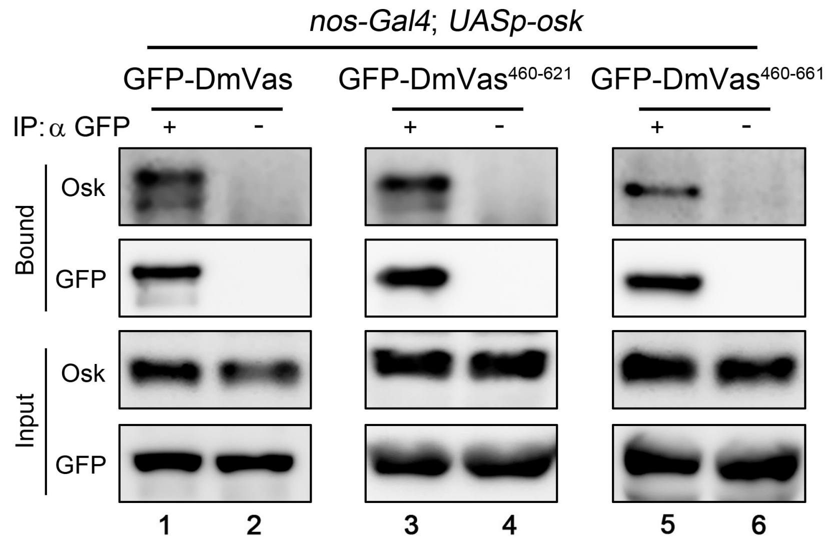

Figure S2. Osk interacts with DmVas$^{460–621/HELICc}$ and DmVas$^{460–661}$

Osk interacts with either full length DmVas, HELICc of DmVas (DmVas$^{460–621/HELICc}$), or HELICc linked with 40 residues at the C-terminal end of DmVas (DmVas$^{460–661}$) in the co-immunoprecipitation (co-IP) experiments. Ovary extracts from adult females co-expressing Osk and GFP-DmVas (Lanes 1,2), Osk and GFP-DmVas$^{460–621/HELICc}$ (Lanes 3,4), or Osk and GFP-DmVas$^{460–661}$ (Lanes 5,6) were immunoprecipitated with anti-GFP antibody. Bound proteins were analysed by Western blotting with anti-Osk or anti-GFP antibodies. + : co-IP with anti-GFP antibody; - : co-IP without anti-GFP antibody (control). Input is 2.5% of the total extract for immunoprecipitation (IP). Genotype of female flies used for ovary extract preparation: *w/w*; *GFP-DmVas/nos-Gal4*; *UASp-osk/+* (Lanes 1,2), *w/w*; *GFP-DmVas$^{460–621/HELICc}$/nos-Gal4*; *UASp-osk/+* (Lanes 3,4), *w/w*; *GFP-DmVas$^{460–661}$/nos-Gal4*; *UASp-osk/+* (Lanes 5,6).

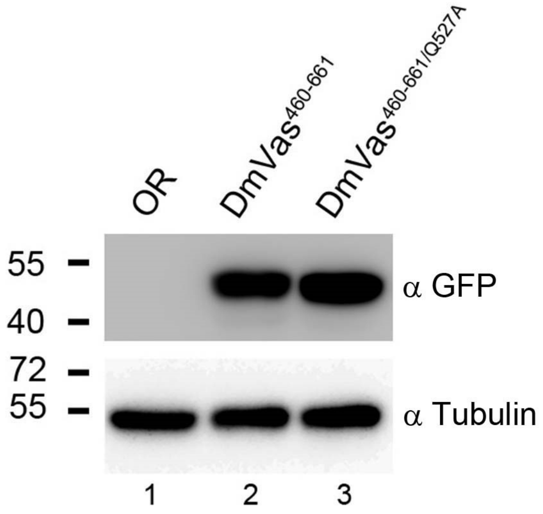

Figure S3. Expression level of green fluorescent protein (GFP)-tagged DmVas$^{460–661}$ and DmVas$^{460–661/Q527A}$

Western blot analyses indicated that the expression levels of GFP-DmVas$^{460–661/Q527A}$ and GFP-DmVas$^{460–661}$ transgenes were similar. Lane 1: wild-type ovary extract (OR), Lane 2: ovary extract from females expressing the GFP-DmVas$^{460–661}$ transgene, Lane 3: ovary extract from females expressing the GFP-DmVas$^{460–661/Q527A}$ transgene. The rabbit anti-GFP antibody was used to detect GFP-tagged *Drosophila* Vasa (DmVas) fusion proteins. The mouse anti-Tubulin antibody was used to detect Tubulin, which served as a loading control.

Supplementary Tables

Table S1. Rescue of *vas* defects in abdominal segment and pole cell formation by GFP-tagged Vasa (Vas) fusion proteins

**Genotypes**	**Number of abdominal segments**				**n**	**Number of pole cells per embryo (±SD)**	**n**
	**0-2**	**3-5**	**6-7**	**8**			
**wild-type (OR)**	0%	0%	0%	100%	392	32.6 (±8.4)	27
vas^{PD}/vas^{PH165}	100%	0%	0%	0%	656	0	30
vas^{PD}/vas^{PH165}; GFP-DmVas/+	0%	0%	0%	100%	567	34.4 (±7.8)	34
vas^{PD}/vas^{PH165}; GFP-DmVas$^{158-661}$/+	0.69%	0%	0%	99.31%	579	20.6 (±6.2)	25
vas^{PD}/vas^{PH165}; GFP-DmVas$^{220-661}$/+	100%	0%	0%	0%	537	0	30
vas^{PD}/vas^{PH165}; GFP-ApD1/+	0%	0.37%	0%	99.63%	543	18.8 (±4.6)	38
vas^{PD}/vas^{PH165}; GFP-A90D/+	0%	0%	0%	100%	593	1.6 (±3.3)	32
vas^{PD}/vas^{PH165}; GFP-ApD2/+	100%	0%	0%	0%	548	0	30

Table S2. Residues located within the predicted protein–protein interaction sites in the helicase superfamily C-terminal (HELICc) domain after 100-ns MD simulations

MD simulations	Sites	Residues
DmVas$^{460–621}$	Site 1	K499, A502, D503, **S518**, I519, **H520**, R523, L524, **Q527**, R528, I544, A545, **T546**
	Site 2	S463, D464, **V465**, K466, Q467, T468, Y470, I484, E487, Q488, H560, G581, R582, N586, **G587**, R588, A589, T590, S612, G613
DmVas$^{460–621/Q527A}$	Site 1	R500, G501, A502, L505, T516, T517, **S518**, **H520**, G521, D522, I544
	Site 2	I484, E487, Q488, N557, I558, K559, H560, N585, N586, **G587**, R588, T590
DmVas$^{470–621}$	Site 1	T516, T517, **S518**, I519, R523, **Q527**, Q530
	Site 2	vanish

Residues previously identified to be involved in Vas localisation are indicated in red colour; the residue Gln527 (Q527) is indicated in blue colour.
